# Robotic-assisted left upper lobe S2 segmentectomy: Identification of vascular and bronchial anatomy

**DOI:** 10.1016/j.xjtc.2025.04.017

**Published:** 2025-05-02

**Authors:** Claire Perez, Lucas Weiser, Kellie Knabe, Raffaele Rocco, Philicia Moonsamy, Andrew R. Brownlee, Harmik J. Soukiasian

**Affiliations:** Department of Surgery, Division of Thoracic Surgery, Cedars Sinai Medical Center, Los Angeles, Calif

**Figure:**
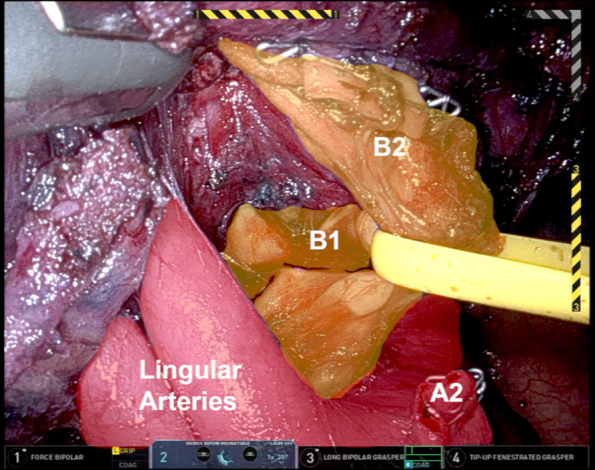
Apical trisegmental bronchus anatomy.

**Central Message**

Indocyanine green may be used to mark the pulmonary lesion and further aid identification of vascular and bronchial anatomy.

Surgical resection remains the mainstay of treatment for early-stage non−small cell lung carcinoma (NSCLC). Traditionally, lobectomy has been the standard surgical approach, but recent trends have highlighted the increasingly important role of segmentectomy, particularly for small-sized peripheral NSCLC. Several studies, including the JCOG0802/WJOG4607L trial, have compared segmentectomy with lobectomy for small-sized tumors and found noninferiority in terms of survival outcomes.^1^ Moreover, the Society of Thoracic Surgeons database has contributed to understanding the importance of patient factors in choosing between lobectomy and sublobar resections, including segmentectomy, showing that the approach depends on tumor characteristics, patient comorbidities, and lymph node involvement.^2^

In recent years, segmentectomy rates have increased.^3^ Notably, there has been a shift toward minimally invasive techniques such as robotic-assisted and thoracoscopic surgery for segmentectomy, leading to reduced postoperative complications and faster recovery.^4^ The growing body of evidence supporting segmentectomy combined with technological advancements suggests that it will play a key and likely evolving role in the treatment of early-stage NSCLC.^5^ In this case report, we describe our approach to a robotic-assisted S2 segmentectomy and highlight the use of indocyanine green (ICG) as a valuable adjunct for identifying the S2 bronchus.

A left upper lobe (LUL) segmentectomy and left lower lobe (LLL) wedge resection were performed in a 77-year-old man with history of prostate cancer and non-Hodgkin lymphoma with incidentally found ground-glass nodules in LUL and LLL. His pulmonary function test was within normal limits. He underwent shape-sensing robotic-assisted bronchoscopy (ssRAB) and biopsy, which demonstrated adenocarcinoma in the LUL and atypia suggestive of carcinoma in the LLL. Final pathology showed 1.5-cm adenocarcinoma in the LUL and 1.2 adenocarcinoma in situ in the LLL. All margins and lymph nodes were negative.

This study was approved by our institutional review board. In addition, the patient consented to the publishing of deidentified pictures and videos collected during this procedure; Cedars-Sinai Medical Center is a teaching hospital, and this practice is standard and included in procedure consent, which was obtained before surgery.

## Technique

### Port Placement and Lesion Marking With ssRAB

The patient is positioned in the right lateral decubitus and intubated with a double-lumen tube (Video 1). This approach streamlines patient positioning for surgical resection. Although this is a more technically challenging method of performing the biopsy or marking, we find that the ssRAB has a flexible tip, which enables navigations through the bronchi seamlessly. Preprocedural computed tomography scan of the chest is reconstructed for the ssRAB system and uploaded into the software, which then processes the data into axial, sagittal, and coronal images before converting them into 3-dimensional renderings of the tracheobronchial tree. Once the lesion is reached, adjunctive methods for localization including 2 and/or 3-dimensional fluoroscopy may be used. The lesion was marked with a 21-g needle biopsy, using a mixture of 0.5 cc ICG and 0.5 cc methylene blue, which was injected slowly to minimize dispersion. Once marking is completed, the patient is then prepped and draped.

A Veress needle is used to insufflate the chest to 10 mm Hg, and an 8-mm robotic port is inserted for access. Three additional ports are placed in the ninth intercostal space, each 8 cm apart. Port 1 is positioned 3 to 4 cm lateral to the spine, port 2 is 8 cm lateral to port 1, and port 3 is 8 cm lateral to port 2, located just posterior to the posterior axillary line. A 12-mm anterior port is placed medially and inferiorly in the seventh interspace, 8 cm from the camera port. A 12-mm robotic-assistant port is positioned inferior to the camera port. The robot is docked, and the camera is centered in the working space.

### LUL Segmentectomy

The lung is retracted anteriorly and apically with a rolled gauze, which exposes the inferior pulmonary ligament. The ligament is then dissected using bipolar cautery. A comprehensive lymph node dissection is carried out as the procedure progresses cephalad. Extensive dissection of the posterior pleura and all lymph nodes in the area is of utmost importance. The lung is then retracted posteriorly to reveal the fissure, which is dissected along the avascular plane of Leriche down to the pulmonary artery: the vessel is exposed in such a manner. A robotic stapler may be used to divide the fissure through the anterior 12-mm port. Given the thickness of the parenchyma within the fissure, bipolar cautery is avoided to reduce the risk of significant postoperative air leaks. A vessel loop can also be used to retract the parenchyma for improved exposure, allowing for completion of the fissure with robotic staplers and providing complete access to the pulmonary artery.

**Figure 1:**
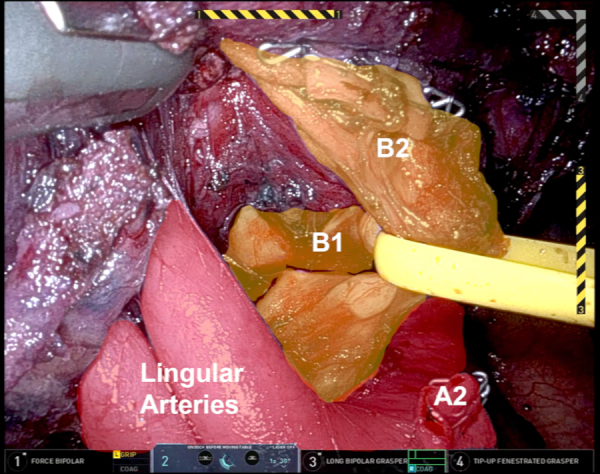
Apical trisegmental bronchus anatomy.

**Figure 2:**
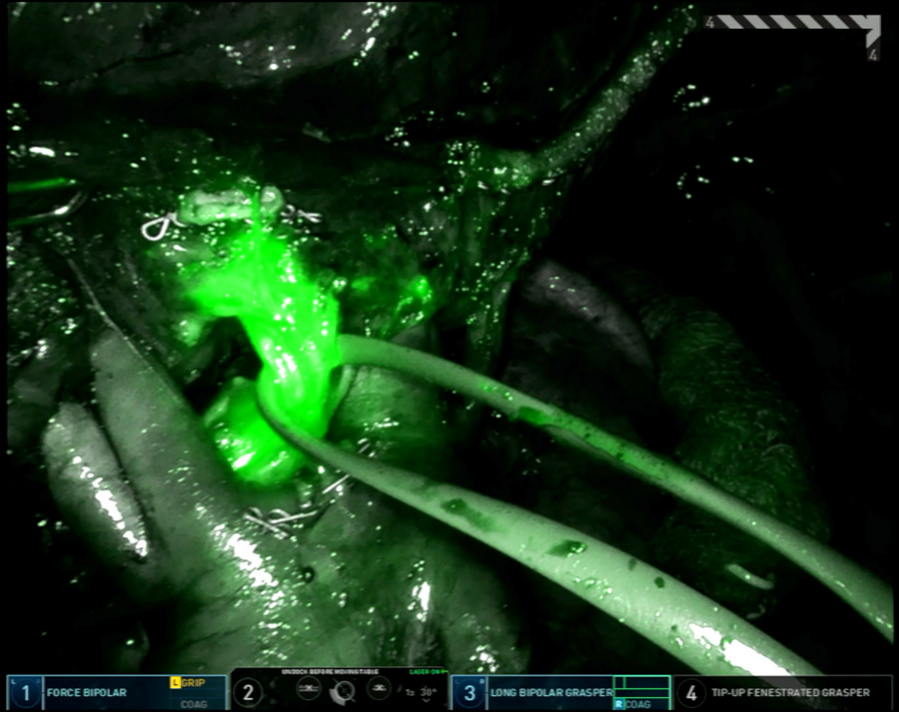
Identification of S2 bronchus using indocyanine green from the marked lesion.

Throughout the procedure, ICG is used to clearly identify the location of the LUL nodule. Once a window is created inferior to the pulmonary artery, a white staple load can be safely used to transect the vessel. The apical trisegmental bronchial anatomy can be difficult to delineate (Figure 1), making it challenging to accurately identify the correct bronchus. However, by using Firefly and tracing the ICG drainage from the marked lesion (Figure 2), the correct bronchus was clearly identified. This was clamped using a stapler, and the left lung was ventilated to ensure that the correct bronchus was isolated before transection. Similarly, the V2 branch was transected using a robotic stapler. Systemic ICG was then administered to outline the devascularized segment and the borders were marked externally to aid in transection using a combination of green and black load staplers.

### LLL Wedge Resection

The LLL nodule, previously marked as well, was identified and meticulously resected using a combination of green and black-load staplers. Firefly mode was again frequently used to confirm adequate margins throughout the procedure. Finally, intercostal nerve blocks were administered for postoperative pain management.

## Comment

Segmentectomy and sublobar resections are becoming more common in the treatment of early-stage NSCLC. In this report, we demonstrate that robotic-assisted LUL segmentectomy, specifically the identification of trisegmental apical bronchial anatomy, can be enhanced by preoperatively lesion marking with ICG. This technique ensures greater precision and safety during the procedure.

## Conflict of Interest Statement

Drs Brownlee and Soukiasian are consultants for Intuitive. All other authors reported no conflicts of interest.

The *Journal* policy requires editors and reviewers to disclose conflicts of interest and to decline handling or reviewing manuscripts for which they may have a conflict of interest. The editors and reviewers of this article have no conflicts of interest.

## Supplementary Data

Video 1 Robotic-assisted S2 segmentectomy and LLL wedge resection. Video available at: https://www.jtcvs.org/article/S2666-2507(25)00164-6/fulltext.
