# Supplementary figures and images for: Robotic-assisted left upper lobe S2 segmentectomy: Identification of vascular and bronchial anatomy
Source: JTCVS Tech. 2025 May 2;31:185–7. doi: 10.1016/j.xjtc.2025.04.017 (PMC12237872; doi:10.1016/j.xjtc.2025.04.017)

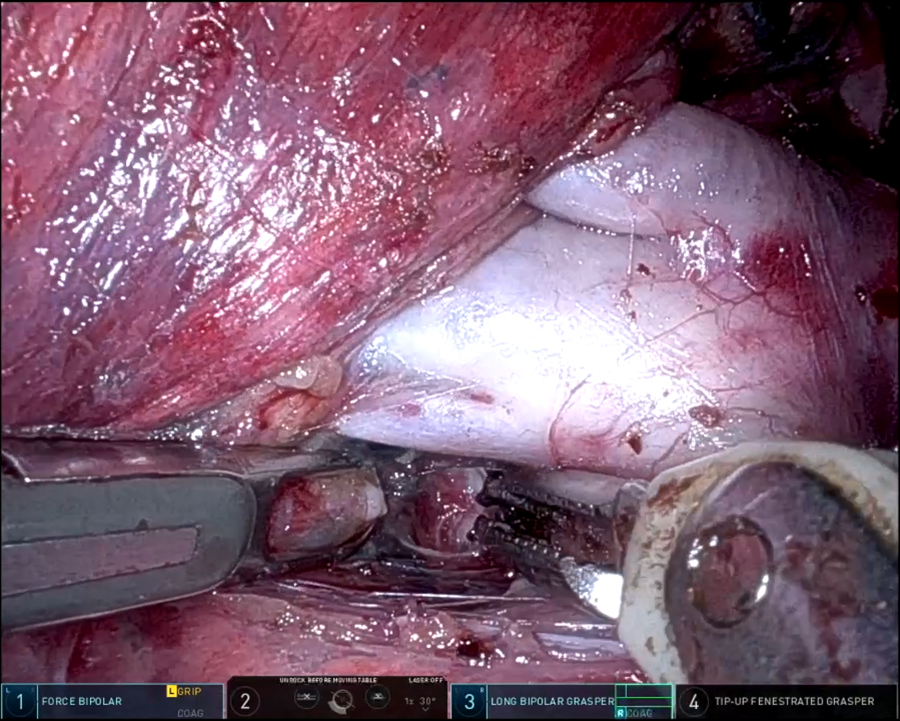

Supplement: Video 1 — Robotic-assisted S2 segmentectomy and LLL wedge resection. Video available at: https://www.jtcvs.org/article/S2666-2507(25)00164-6/fulltext. [file fx2.jpg]
